# Trends and comparisons of diabetes burden in China and the world from 1990 to 2021,with forecasts to 2050:a systematic analysis of the global burden of disease study 2021

**DOI:** 10.1186/s13098-025-01885-4

**Published:** 2025-08-02

**Authors:** Chenqing Liu, Yun Li, Nan Wang, Yandong Wu, Jiaqi Liu, Mengjie Ding, Shuhan Liu, Yibing Hao, Ying Wu, Shaopeng Liu

**Affiliations:** 1 School of Public Health, North China University of Science and Technology, Tangshan, 063210 China; 2 School of Public Health, Wuhan University of Science and Technology, Wuhan, 430000 China; 3 National International Cooperation Base for Science and Technology On Geriatric Medicine, North China University of Science and Technology, Tangshan, 063210 China

**Keywords:** Disease burden, Diabetes, Global burden of disease, Public health

## Abstract

**Background:**

Diabetes is a major global public health issue. This study investigated the trends in the age—and gender—specific burden of diabetes in China and worldwide from 1990 to 2021, and predicted the prevalence of diabetes in 2050.

**Methods:**

Using publicly available data from the Global Burden of Disease (GBD) database from 1990 to 2021, we comprehensively applied Joinpoint regression and age—period—cohort (APC) analysis to reveal the epidemiological characteristics, conducted decomposition analysis to identify the driving factors of burden changes, and used the autoregressive integrated moving average (ARIMA) model to project the disease burden of diabetes from 2022 to 2050.

**Results:**

From 1990 to 2021, both in China and globally, the age—standardized incidence rate (ASIR), age—standardized prevalence rate (ASPR), and age—standardized disability—adjusted life year rate (ASDR) of diabetes showed an upward trend. In contrast, China's age—standardized mortality rate (ASMR) of diabetes decreased, while the global ASMR increased. The average annual percentage changes (AAPC) of China's ASIR, ASPR, ASMR, and ASDR were 1.29, 1.76,− 0.30, and 0.76% respectively, compared with 1.74, 2.10, 0.25, and 1.05% for the global diabetes burden.

**Conclusions:**

In China, the incidence, prevalence, and Disability-Adjusted Life Years (DALYs) of diabetes increased, while the mortality rate decreased. It is projected that by 2050, the number of diabetes patients in China will reach 84.01 million, and globally, it will reach 1038.2 million. Given China's large population and the trend of population aging, it is essential to formulate targeted prevention and control strategies to address the challenge of diabetes.

**Supplementary Information:**

The online version contains supplementary material available at 10.1186/s13098-025-01885-4.

## Background

Diabetes has become one of the core challenges in global health in the twenty-first century, and its disease burden is growing at an alarming rate. According to the GBD 2021 study published by The Lancet in 2024, the global number of people with diabetes increased from 139 million in 1990 to 526 million in 2021, with an ASPR that has been continuously rising at an annual rate of 2.10% [1]. China, as the country with the heaviest burden of diabetes globally, had 117 million patients in 2021, accounting for 22% of the global diabetes population. The ASPR in China rose from 3581.17/100,000 in 1990 to 6142.29/100,000, with a year-on-year growth rate of 1.77%, highlighting the urgency of the prevention and control situation [1, 2].

The dynamic evolution of epidemiological driving factors has attracted much attention in recent years. A study in Diabetes Care in 2023 pointed out that the increase in diabetes burden in China is closely related to the Westernization of lifestyle. During the period from 1990 to 2021, the consumption of sugary beverages increased by 4.30% annually, the rate of physical inactivity reached 31.70%, leading to a sharp increase in the obesity rate from 3.10 to 16.40%, and the risk of diabetes in obese individuals was 3.20 times that of normal-weight individuals [3, 4]. At the same time, population aging contributed 32% to the increase in ASPR, which is consistent with the conclusion of a study in Geriatrics and Clinical Medicine in 2022 that “aging leads to a 15% decrease in insulin sensitivity every ten years” [5]. The unique social and economic transformation in China has profoundly shaped the characteristics of diabetes prevalence. A study in the Journal of the American Medical Association Sub-press in 2023 showed that during the process of urbanization from 26.40 to 64.70%, the ASPR growth rate in rural areas (1.92% per year) exceeded that in urban areas (1.58% per year), attributed to the widespread consumption of high-fat diets and the insufficient accessibility of medical resources in rural areas [6, 7]. This urban–rural disparity was particularly significant between the eastern coastal regions (ASIR 266.48/100,000) and the less developed western regions (223.72/100,000), reflecting the complex relationship between economic development levels and disease burden [1].

Although institutions such as the International Diabetes Federation (IDF) have conducted numerous studies, there are still significant knowledge gaps in three aspects: Firstly, the analysis of long-term trends lacks dynamic segmentation—existing studies mostly focus on a single year or short periods, for example, the IDF 2021 report only covers up to 2020 and fails to systematically analyze the ASPR of diabetes ASPR during this period with a growth rate of 4.69% [2, 8]; Secondly, the prediction models do not incorporate cohort effects, the IDF 2021 prediction of 83 million diabetes patients in China in 2050 did not fully consider the differences in birth cohorts; Thirdly, the heterogeneity mechanisms of gender and age have not been clarified. These research gaps make it difficult for the existing evidence to meet the practical needs of “Healthy China 2030” for precise prevention and control of chronic diseases.

This study, based on the full-cycle data from the GBD 2021 database, integrates spatio-temporal dynamic analysis and cohort effect prediction. The aim is to analyze the phased evolution characteristics of diabetes burden in China through Joinpoint regression; using the APC model to separate the independent contributions of aging, period effects, and birth cohorts; Combining the ARIMA model to generate dynamic predictions that take into account social and economic transitions. The research results will provide key support for China to formulate “evidence-based and population-oriented” diabetes prevention and control strategies, especially in the context of accelerating aging, providing scientific basis for the allocation of medical resources and the setting of intervention measures priorities [1, 9].

## Methods

### Data sources

This study utilizes data from the GBD 2021 data base curated by the Institute for Health Metrics and Evaluation (IHME) [1].The GBD divides the world into different regions and super-regions. In the “about GBD”section of the GBD website, the subsection “which locations are studied in the GBD”clearly states that the GBD regions are divided based on two criteria: epidemiological similarity and geographical proximity. The world is divided into 21 regions and 7 super-regions, namely high-income regions, Latin America and the Caribbean region, sub-Saharan Africa region, North Africa and the Middle East region, Southeast Asia, East Asia and Oceania region, South Asia region, Central Europe, Eastern Europe and Central Asia region. China belongs to the East Asia region and is located within the Southeast Asia, East Asia and Oceania super-region. The countries and regions within this super-region share certain similarities in terms of disease prevalence characteristics, health status, and health resources. As a cornerstone of global health research, the GBD initiative systematically assesses the comprehensive impact of diseases on population health worldwide by quantifying key indicators such as incidence, prevalence, mortality rate, and DALYs. Led by IHME, it integrates multi-source data from 204 countries and territories spanning 1990 to 2021, providing sex- and age-stratified estimates with 95% uncertainty intervals (UI) for 371 diseases and injuries—including incidence, prevalence, years lived with disability (YLD), years of life lost (YLL), and DALYs [10]. The 95% UI, generated through 1000 Monte Carlo samplings, quantifies the overall uncertainty arising from data gaps and model assumptions, offering a more comprehensive reflection of result reliability compared to traditional confidence intervals (CI) [11, 12].

Data processing and estimation methodologies have been comprehensively documented in recent GBD studies. Modeling employed dual computational frameworks: DisMod-MR—a Bayesian meta-regression platform ensuring internal consistency through synthesis of epidemiological parameters (incidence, prevalence, remission, mortality) [1, 13]; and CODEm—an ensemble modeling architecture specialized for mortality analysis, which optimizes covariate selection via predictive validity testing [13, 14].

From the GBD database, we extracted the incidence, prevalence, mortality, DALYs, and their corresponding age-standardized rates—including ASIR、ASPR、ASMR and ASDR—for diabetes in China and globally from 1990 to 2021. Age-standardized rates adjust disease indicators to a unified age structure, eliminating the influence of differences in population age composition on results and facilitating valid comparisons across regions or time periods [1, 2]. Additionally, crude incidence rate (CIR), crude prevalence rate (CPR), crude mortality rate (CMR), and crude DALY rate (CDR) were used to evaluate the diabetes burden.

This study was exempt from ethics committee approval as it utilized publicly accessible, de-identified population-level data from the Global Health Data Exchange (GHDx) and its affiliated tools (http://ghdx.healthdata.org/gbd-results-tool) [14], in full compliance with the Guidelines for Accurate and Transparent Health Estimates Reporting (GATHER).

### Statistical analyses

Descriptive analysis was used to describe the global burden of diabetes in China and the world by sex, age group and region from 1990 to 2021.Joinpoint software (National Cancer Institute, USA) was used to calculate the AAPC and 95% confidence interval (95% CI) to determine the trend of diabetes burden [15].Joinpoint regression, proposed by Kim et al. in 1998 [16], is a key statistical method in time trend analysis for evaluating disease prevalence or mortality trends over time, and in this study, it precisely identifies and quantifies significant change points in time series data of diabetes incidence, prevalence, mortality, and DALYs in China and globally [17]. Its core idea is to establish segmented regression by dividing the study period into intervals at multiple joinpoints, fitting and optimizing trends for each interval to assess disease change characteristics across different time ranges [16]. Optimal joinpoints are determined via the grid search method (GSM), with 95% CIs derived from Monte Carlo permutation tests [18]; this involves optimizing segmented fitting by gridding the study period, calculating mean square error (MSE) for each scenario, and generating 2.5 and 97.5% percentiles of the parameter distribution to construct CIs [12]. CI width reflects trend robustness: narrow CIs indicate stable incidence trends, while wide CIs suggest fluctuations from factors like regional differences, requiring further verification, thus providing rigorous statistical support for comparing temporal changes in diabetes burden between China and the globe. Joinpoint software (National Cancer Institute, USA) was used to calculate the AAPC—the average annual change trend of disease indicators via Joinpoint regression to assess long-term time series growth or decline rates and 95% CIs to determine diabetes burden trends [15], using the least square method to fit prevalence changes and overcome subjectivity in traditional linear trend analysis, with turning points identified by residual sum of squares and significance judged by comparing AAPC with 0 (P < 0.05) [8].

The APC model—used to decompose the influence of age, period, and birth cohort on disease trends, performing separate analysis of independent effects of each dimension using log-linear regression [8]—was used to analyze the effects of age, period and cohort factors on the incidence or mortality of diabetes. Based on Poisson distribution, the model was analyzed by log-linear regression model $\mathit{log}(Yi)=\mu +\alpha *{ age }_{i}+\beta *{ period }_{i}+\gamma *{ cohort }_{i}+\varepsilon$, where $Yi$ represents the prevalence or mortality of diabetes, $\alpha$,$\beta$ and $\gamma$ are the coefficients of age, period and cohort, and $\mu$ is the model intercept. $\varepsilon$ is the residual [16, 21]. The intrinsic estimator (IE) method built into the model was used to obtain the net effects of the three dimensions.

Decomposition analysis was used to visually show the role of aging, demographic and epidemiological factors in the changes in diabetes incidence, prevalence, mortality and DALYs from 1990 to 2021 [20]_._ ARIMA model was used to predict the trend of diabetes prevalence from 2022–2050 [21]_._ The model consists of autoregressive (AR) and moving average (MA) components, which assume that the autocorrelation of data series can be represented by the model, so as to predict future trends based on historical data. The equation is $Yt ={\varphi }_{1}{Y}_{t-1}+{\varphi }_{2}{Y}_{t-2}+\dots +{\varphi }_{t-p}{Y}_{t-p}+ et -{\theta }_{1}{e}_{t-1}-\cdots -{\theta }_{q}{e}_{t-q}$, where (${\varphi }_{1}{Y}_{t-1}+{\varphi }_{2}{Y}_{t-2}+\dots +{\varphi }_{t-p}{Y}_{t-p}+ et$) is the MA model part,${Y}_{t-p}$ is the observed value at the period of (t-p), p and q represent the model order of AR and MA, and et is the random error at the period of t [21].When using this model, the data sequence is required to be a stationary random sequence with zero mean [22].

With the help of R statistical software (version 4.4.2) and Joinpoint software (version 5.3.0), statistical analysis and visual display of data were completed [14].

## Results

### Burden of diabetes in China and incidence of diabetes worldwide

In terms of incidence, the number of diabetes cases in China increased from 1,836,953 cases (95% CI 1,648,300–2,052,190) in 1990 to 4,003,544 cases (95% CI 3,603,717–4,441,554) in 2021, showing a cumulative increase of 117.94%. Globally, however, incident cases rose from 7,780,272 cases (95% CI 7,193,591–8,397,763) in 1990 to 24,442,180 cases (95% CI 22,643,513–26,301,698) in 2021, with a cumulative growth of 214.16%. The ASIR in China increased from 163.34 per 100,000 population (95% CI 144.74–181.98) in 1990 to 244.57 per 100,000 population (95% CI 223.72–266.48) in 2021. Globally, the ASIR rose from 167.65 per 100,000 population (95% CI 153.97–181.03) in 1990 to 287.31 per 100,000 population (95% CI 266.94–308.84) in 2021. Meanwhile, the AAPC in incidence rates from 1990 to 2021 was 1.30% (95% CI 1.19–1.40) in China and 1.74% (95% CI 1.68–1.81) globally (Table 1).

**Table 1:** Diabetes-related indicators in China and the world from 1990 to 2021

Location	Measure	1990		2021		1990-2021AAPC
		All-ages cases	Age-standardized rates per 100,000 people	All-ages cases	Age-standardized rates per 100,000 people	
		n (95% CI)	n (95% CI)	n (95% CI)	n (95% CI)	n (95% CI)
China	Incidence	1836953 (1648300–2052190)	163.34 (144.74–181.98)	4003544 (3603717–4441554)	244.57 (223.72–266.48)	1.30* (1.19–1.40)
	Prevalence	35352299 (31762275–39043129)	3581.17 (3197.11–3968.53)	117288554 (107649694–128071008)	6142.29 (5601.11–6704.38)	1.77* (1.63–1.90)
	Deaths	72191 (63382–82129)	9.83 (8.64–11.08)	178476 (147957–211655)	8.98 (7.45–10.61)	− 0.24 (− 0.49–0.01)
	DALYs	4288784 (3513609–5227037)	466.90 (385.22–561.90)	11713614 (9046222–15013010)	585.43 (448.94–754.32)	0.76 (0.65–0.87)
Global	Incidence	7780272 (7193591–8397763)	167.65 (153.97–181.03)	24442180 (22643513–26301698)	287.31 (266.94–308.84)	1.74* (1.68–1.81
	Prevalence	139107877 (128055501–150839975)	3215.75 (2962.07–3498.90)	525654113 (490915907–565380792)	6123.59 (5723.41–6585.82)	2.10* (2.02–2.18)
	Deaths	672022 (635444–705306)	18.17 (17.03–19.07)	1656635 (1537699–1759551)	19.61 (18.12–20.83)	0.26* (0.16–0.36)
	DALYs	27504001 (24299172–31667085)	663.11 (587.04–761.50)	78938587 (66772201–94495830)	916.25 (775.93–1096.15)	1.06* (0.99–1.13)

### Burden of diabetes in China and prevalence of diabetes worldwide

In terms of prevalence, the number of diabetes cases in China increased from 35,352,299 cases (95% CI 31,762,275–39,043,129) in 1990 to 117,288,554 cases (95% CI 107,649,694–128,071,008) in 2021, showing a cumulative growth of 231.77%. Globally, however, prevalent cases rose from 139,107,877 cases (95% CI 128,055,501–150,839,975) in 1990 to 525,654,113 cases (95% CI 490,915,907–565,380,792) in 2021, with a cumulative increase of 277.87%. The ASPR in China increased from 3581.17 per 100,000 population (95% CI 3197.11–3968.53) in 1990 to 6142.29 per 100,000 population (95% CI 5601.11–6704.38) in 2021. Globally, the ASPR rose from 3,215.75 per 100,000 population (95% CI 2,962.07–3,498.90) in 1990 to 6123.59 per 100,000 population (95% CI 5723.41–6585.82) in 2021. From 1990 to 2021, the AAPC in prevalence rates was 1.77% (95% CI 1.63–1.90) in China and 2.10% (95% CI 2.02–2.18) globally (Table 1).

### Burden of diabetes in China and mortality of diabetes worldwide

Globally, diabetes caused 1,656,635 deaths in 2021 (95% CI 1,537,699–1,759,551), representing a 146.51% increase compared to 1990. In China, the mortality rate increased by 147.22% from 1990 to 2021.The global ASMR rose from 18.17 per 100,000 population (95% CI 17.03–19.07) in 1990 to 19.61 per 100,000 population (95% CI 18.12–20.83) in 2021. In contrast, China’s ASMR declined from 9.83 per 100,000 population (95% CI 8.64–11.08) in 1990 to 8.98 per 100,000 population (95% CI 7.45–10.61) in 2021. From 1990 to 2021, the AAPC in mortality rates globally increased by 0.26% (95% CI 0.16–0.36), while in China, it decreased by 0.24% (95% CI − 0.49–0.01) (Table 1).

### DALYs of diabetes in China and globally

In China, the DALYs due to diabetes were 4,288,784 (95% CI 3,513,609–5,227,037) in 1990 and rose to 11,713,614 (95% CI 9,046,222–15,013,010) in 2021, representing a 173.12% increase compared to 1990. Globally, DALYs increased by 187.01% from 1990 to 2021.The ASDR in China increased from 466.90 per 100,000 population (95% CI 385.22–561.90) in 1990 to 585.43 per 100,000 population (95% CI 448.94–754.32) in 2021. Globally, the ASDR rose from 663.11 per 100,000 population (95% CI 587.04–761.50) in 1990 to 916.25 per 100,000 population (95% CI 775.93–1096.15) in 2021.From 1990 to 2021, the AAPC in DALYs was 0.76% (95% CI 0.65–0.87) in China and 1.06% (95% CI 0.99–1.13) globally (Table 1).

### Joinpoint regression analysis of the burden of diabetes in China and worldwide

**Fig. 1:**
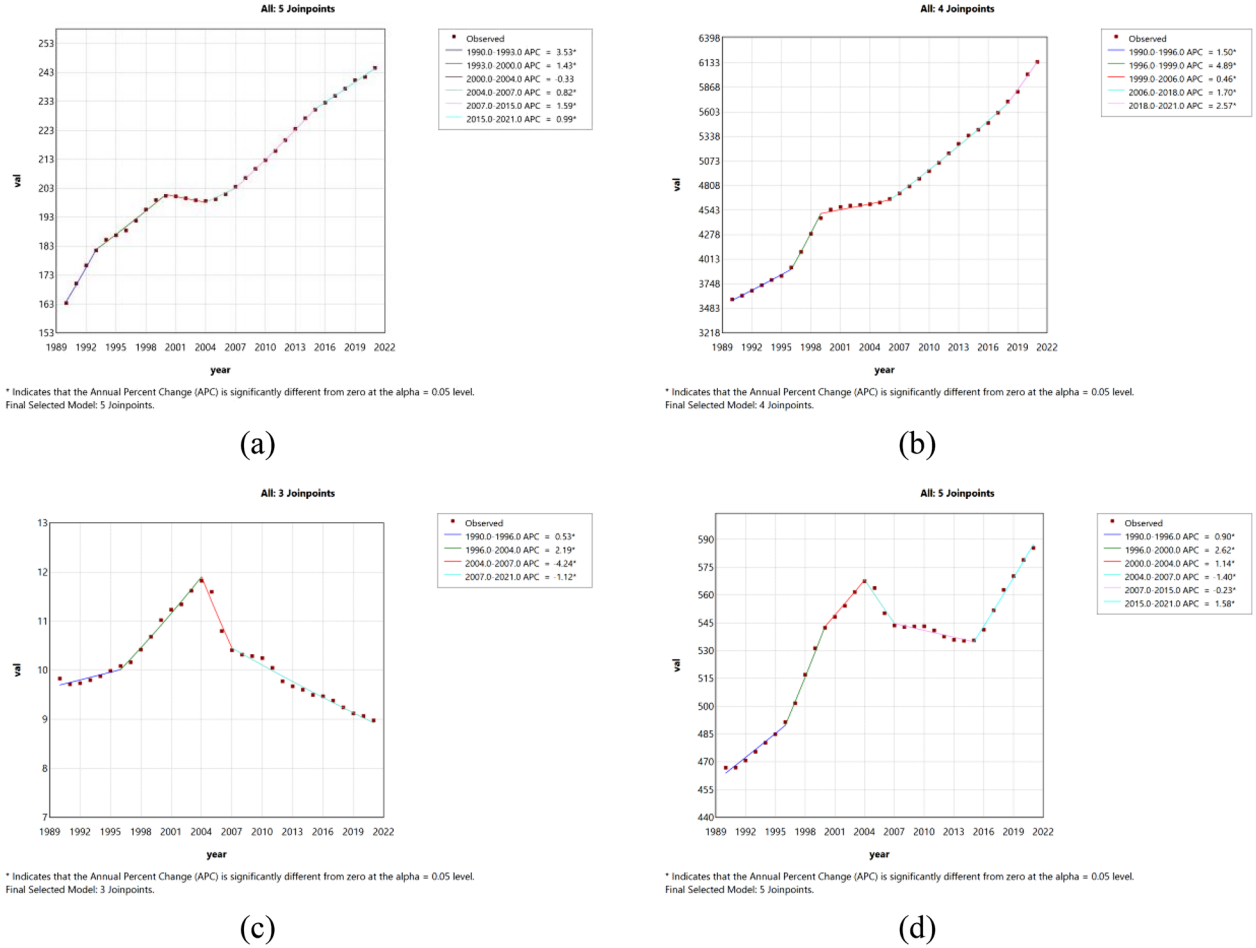
The APC of ASIR, ASPR, ASMR, and ASDR of diabetes in China from 1990 to 2021 (*means p-values < 0.05 and significant results). (**a**) ASIR; (**b**) ASPR; (**c**) ASMR; (**d**) ASDR

**Fig. 2:**
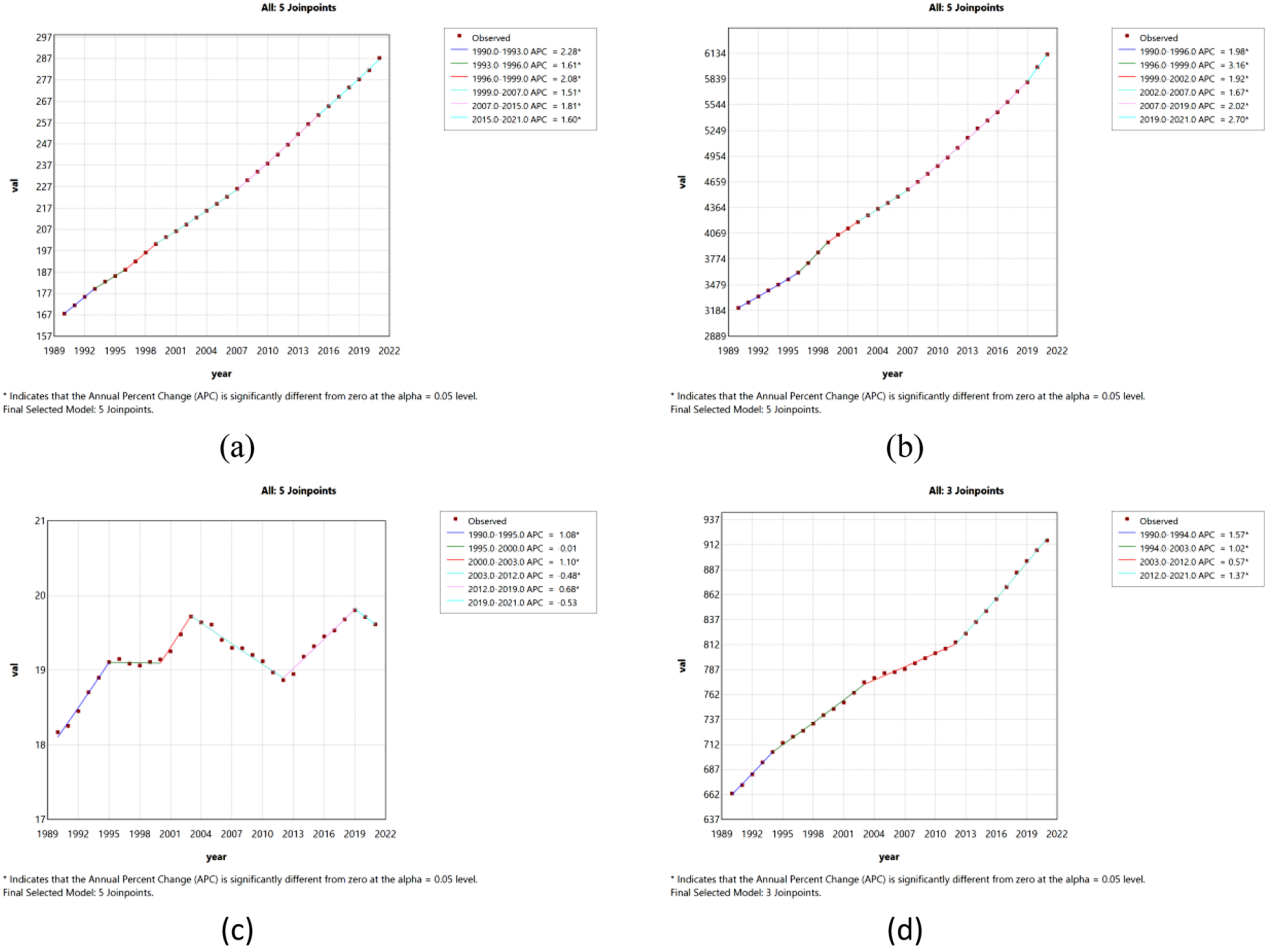
The APC of ASIR, ASPR, ASMR, and ASDR of diabetes in Global from 1990 to 2021 (*means p-values < 0.05 and significant results). (**a**) ASIR; (**b**) ASPR; (**c**) ASMR; (**d**) ASDR

The Joinpoint regression analysis of ASIR, ASPR, ASMR, and DALYs for diabetes in China and worldwide from 1990 to 2021 is depicted in Figs. 1 and 2. The annual percentage change (APC) of ASIR for diabetes in China showed an overall upward trend from 1990 to 2021 (ASIR: 1990–1993 APC 3.53, p < 0.05; 1993–2000 APC 1.41, p < 0.05; 2004–2007 APC 0.82, p < 0.05; 2007–2015, APC 1.59, p < 0.05; 2015–2021, APC 0.99, p < 0.05), but there was a downward trend from 2000 to 2004 (APC  − 0.33). The APC of diabetes ASPR in China showed an overall upward trend from 1990 to 2021 (ASPR 1990–1996 APC 1.52, p < 0.05; 1996–1999 APC 4.69, p < 0.05; 1999–2002 APC 0.82; 2002–2006 APC 0.26; 2006–2018 APC 1.72, p < 0.05; 2018–2021 APC 2.54, p < 0.05). Globally, ASIR and ASPR showed a significant upward trend (p < 0.05). China's ASMR showed a significant upward trend from 1992 to 2004 (p < 0.05), and showed a downward trend in the other years. From 1990 to 2021, ASMR on a global scale showed a fluctuating trend of rising and falling, rising and falling. The global ASDR showed an upward trend, while the ASDR in China showed a significant downward trend from 2004 to 2015 (p < 0.05).

### Trends in the burden of diabetes in China and worldwide

**Fig. 3:**
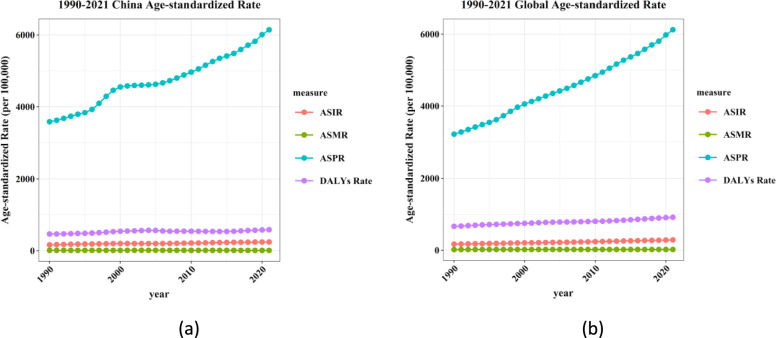
Trend comparison of ASIR, ASPR, ASMR, and ASDR of diabetes in China and worldwide from 1990 to 2021

From 1990 to 2021, China's ASPR exhibited a consistent upward trend, progressively increasing from a relatively low baseline in 1990 to a significantly higher value in 2021. In contrast, the ASMR, ASIR, and DALYs Rate remained largely stable during this period. Globally, the trend of ASPR mirrored that of China, demonstrating a marked upward trajectory with continuous growth observed from 1990 to 2021. The global DALYs Rate was relatively stable, albeit with a slight upward inclination, which aligns with the trend observed in China. The ASIR showed minimal variation, maintaining relative stability throughout the period. Similarly, the ASMR remained at a consistent level, indicating that the global ASMR did not undergo significant fluctuations (Fig. 3).

### Gender disparities in the burden of RHD in different age groups in China and Global

**Fig.4:**
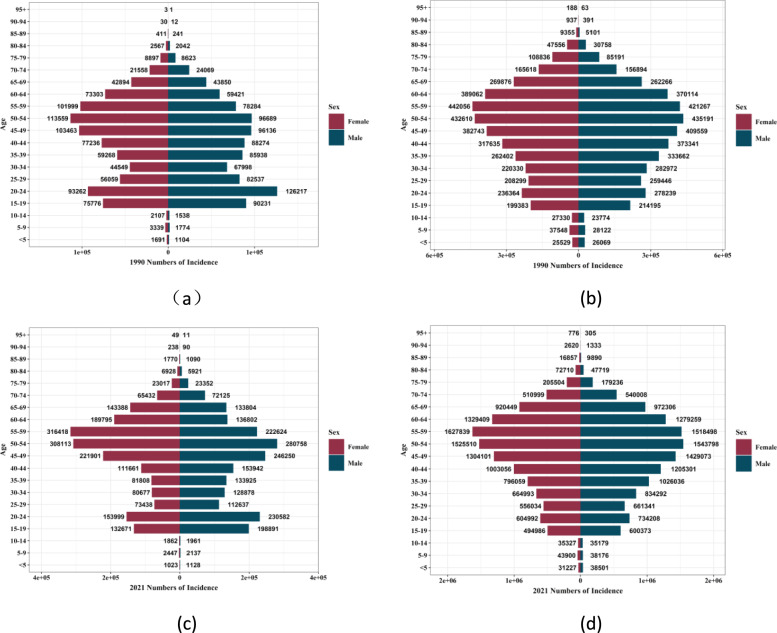
Incidence of diabetes in different age groups for men and women in China and globally, 1990 and 2021 (**a**) Incidence in China in 1990; (**b**) Global incidence in 1990; (**c**) incidence in China, 2021; (**d**) Global incidence, 2021

**Fig.5:**
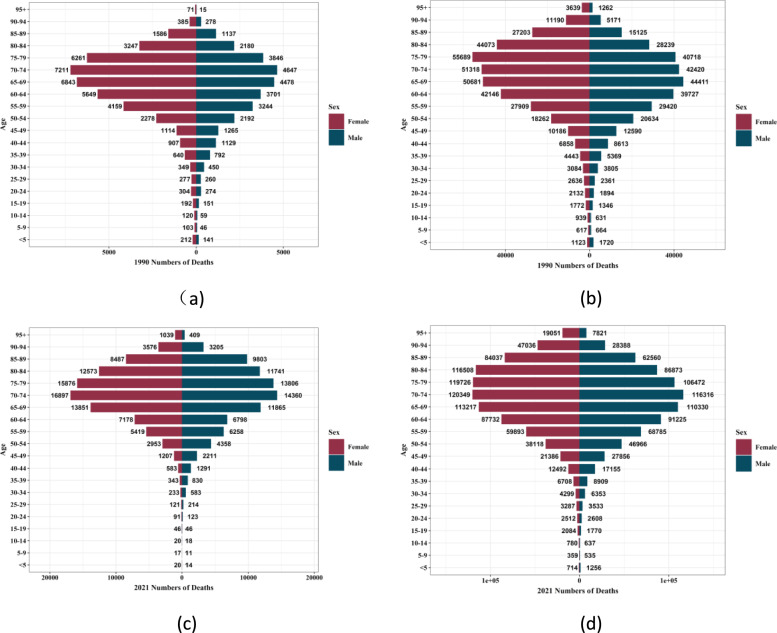
Mortality rates of diabetes among men and women of different age groups in China and globally in 1990 and 2021 (**a**) Mortality in China in 1990; (**b**) Global mortality in 1990 (**c**) Mortality in China in 2021 (**d**) Global mortality in 2021

**Fig.6:**
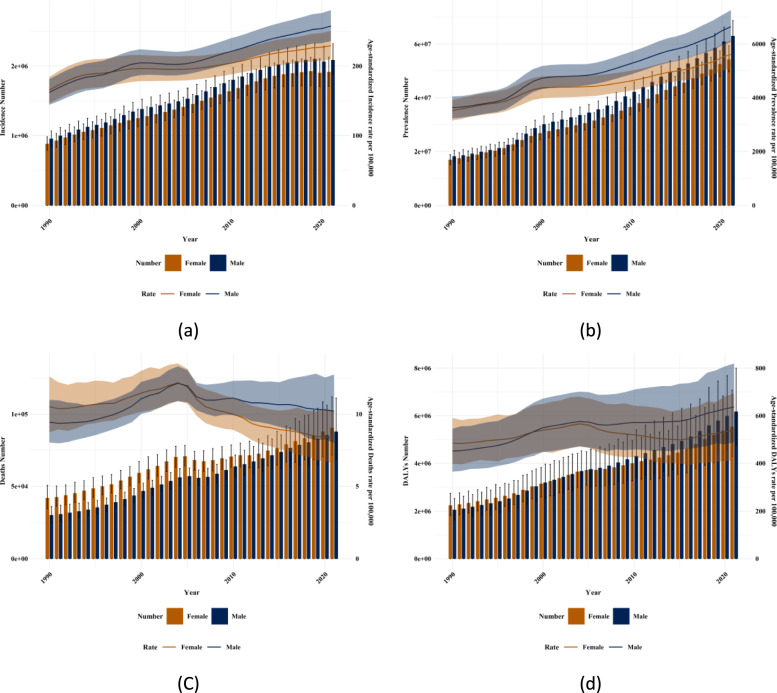
Comparison of full-age cases and age-standardized rates of incidence, prevalence, mortality and DALYs among men and women in China from 1990 to 2021. (**a**) Incident cases and ASIR; (**b**) Prevalent cases and ASPR; (**c**) Death cases and ASMR; (**d**) DALYs counts and ASDR. Bar charts represent counts; lines represent age-standardized rates

Figures 4 and 5, as well as Supplementary Figs. 1 and 2 show the incidence, prevalence, mortality rate, and DALYs of diabetes among men and women in different age groups in China and globally in 1990 and 2019. In China, in 1990, in terms of the incidence, prevalence, mortality rate, and DALYs of diabetes, all showed a trend of reaching a peak in the middle and old age stages with the increase of age. In terms of gender, the indicators of men were higher than those of women in most dimensions, but in some older age groups, the indicators of women were higher than those of men in some aspects. In 2021, in all dimensions of diabetes indicators, the middle and old age stages (especially the age group of 65–94 years old) were all at a relatively high level, and men were higher than women in most dimensions such as the incidence rate, mortality rate, and DALYs. Globally (Supplementary Figs. 1 and 2), the trends of the incidence rate, prevalence, mortality rate, and DALYs were consistent with those in China. The incidence rate and prevalence showed an overall upward trend in all age groups, especially in the middle and old age stages; the mortality rate and DALYs increased with the increase of age and were at a relatively high level in the older age groups. In terms of gender, in most dimensions, the indicators of men in most age groups were higher than those of women, but in some older age groups, the indicators of women were higher than those of men in some aspects. Figure 6 and Supplementary Fig. 3 show the comparison of the disease burden of diabetes and the age-standardized rates among men and women of all age groups in China and globally from 1990 to 2021. During the period from 1990 to 2021 in China, in terms of the incidence rate, prevalence, the number of DALYs, and the number of deaths of diabetes, both men and women showed an overall upward trend. In terms of the age-standardized rates, the incidence rate, prevalence, and DALYs rate increased, and the change of the mortality rate was relatively complex but higher in men than in women. Overall, men were relatively more affected by diabetes in all dimensions. Globally (Supplementary Fig. 3), the overall change trend was consistent with that in China.

### Age, period and cohort effects on diabetes prevalence and mortality

**Fig.7:**
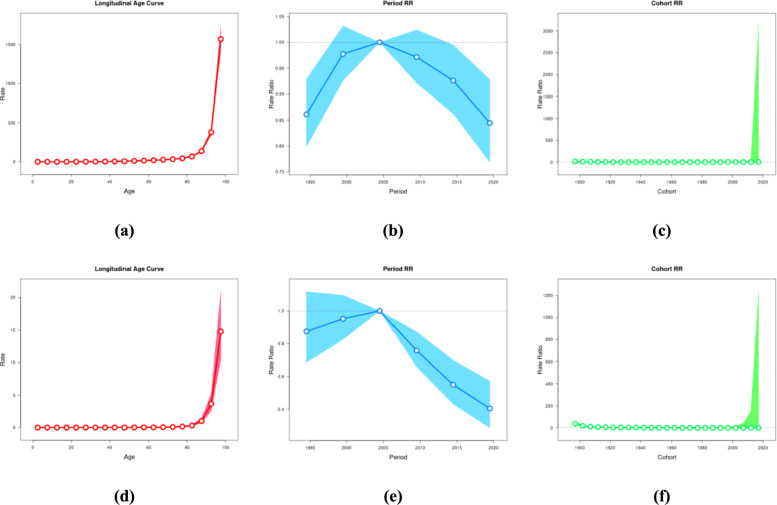
Trends in the prevalence and mortality of diabetes in China from 1990 to 2021. (**a**) age effect of prevalence (**b**) period effect of prevalence (**c**) cohort effect of prevalence (**d**) age effect of mortality (**e**) period effect of mortality (**f**) cohort effect of mortality

The age-period-cohort effects of diabetes incidence and mortality in China and the world are presented in Fig. 7 and Supplementary Fig. 4. In GBD2021, the prevalence of diabetes was defined as the percentage of people with diabetes in the total population, and the mortality rate of diabetes was defined as the number of deaths due to diabetes per 100,000 population. To run the age-period cohort model, we divided the data series into consecutive 5 year intervals from 1992 to 2021. Data from 1990, 1991 were not analyzed because they did not span the 5 year interval. Age was also divided into 5 year age groups ranging from 0 to 95 + years.

The longitudinal age curve of the prevalence in China (Fig. 7a) was around the age of 0–80 years, and the prevalence was low and changed slowly. However, after the age of 80 years, the rate began to rise rapidly and reached an extremely high value near the age of 100 years, indicating that the rate showed a sharp upward trend with aging, especially at advanced ages. The period relative risk rate of China's prevalence (Fig. 7b), setting the 2002–2006 cohort as the standard 1, and comparing the other cohorts with this cohort, it can be seen from the figure that during 1995–2005, the rate ratio showed an upward trend and reached a peak (about 1.02). From 2005 to 2020, the rate ratio showed a downward trend and decreased to a low level (about 0.85) in 2020, p < 0.05 was statistically significant. Cohort relative risk rates for prevalence in China (Fig. 7c), with the 1957–1961 cohort set as standard 1 and the remaining cohorts compared with this, p < 0.05 was statistically significant. In most of the cohort, the rate ratios were close to zero, low and unchanged. However, towards 2020, a very high rate ratio value appeared, indicating that the relative risk rate of the birth cohort around 2020 increased greatly compared with other years. The longitudinal age curve of mortality in China (Fig. 7d) showed the same trend as that of the longitudinal age curve of prevalence in China. The period relative risk rate of mortality in China (Fig. 7e), setting the 2002–2006 cohort as the standard 1, and comparing the other cohorts with this cohort, the figure shows that the rate ratio increased from 1995 to 2005, and decreased from 2005 to 2020. By 2020, it decreased to about 0.4, p < 0.05 was statistically significant; The trend of cohort relative risk rates for mortality in China (Fig. 7f) was basically consistent with that of cohort relative risk rates for disease in China.

### Decomposition analysis of gender specific incidence, prevalence, mortality and DALYs of diabetes in China, 1990–2021

**Fig.8:**
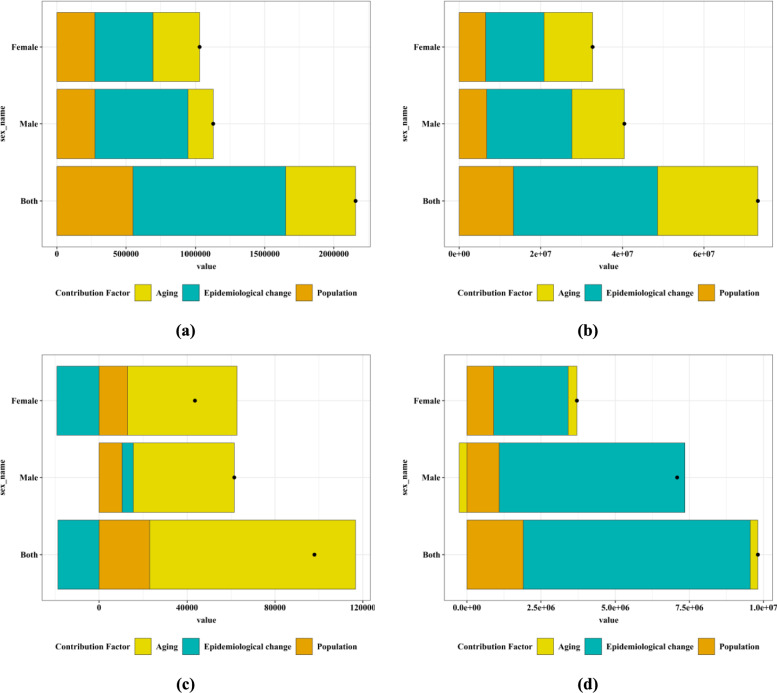
Decomposition analysis on the incidence, prevalence, mortality and DALYs of diabetes by gender in China, 1990–2021 (**a**) incidence (**b**) prevalence (**c**) mortality (**d**). DALYs, black dots indicate the overall change value of the contribution of all 3 components

Through the decomposition analysis, as shown in Fig. 8, the roles of the three factors (eg., aging, population, and epidemiological change) that drove the changes in the incidence rate, prevalence rate, mortality rate, and DALYs from 1990 to 2021 are intuitively presented. From 1990 to 2021, for each component, the magnitude of the positive value indicates the corresponding increase in the incidence rate, prevalence rate, mortality rate, and DALYs of diabetes attributed to that component; the magnitude of the negative value indicates the corresponding decrease in the incidence rate, prevalence rate, mortality rate, and DALYs due to the relevant component. As can be seen from Fig. 8, for the incidence rate and prevalence rate, the changes in aging, population, and epidemiology can all lead to an increase in the risk of diabetes, among which the change in epidemiology accounts for the largest proportion; for the mortality rate, aging accounts for the largest proportion, and the change in epidemiology will cause a decrease in the risk of diabetes mortality in women (-35.42%); for DALYs, the change in epidemiology accounts for the largest proportion, and aging will cause a decrease in the DALYs of diabetes in men (− 3.17%).

### Prediction of diabetes prevalence in China and the world in 2022–2050

**Fig.9:**
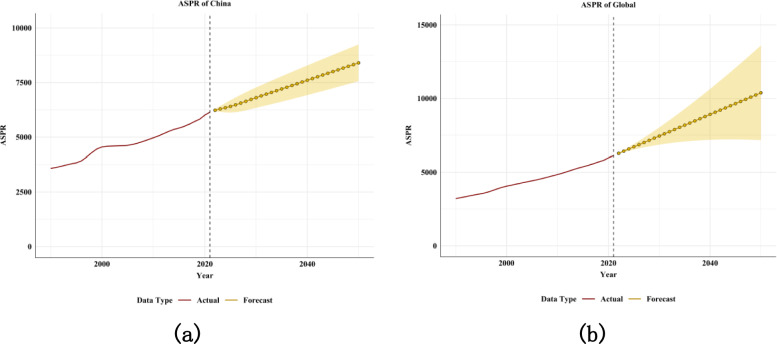
Prediction of diabetes prevalence in China and the world from 2022–2050. The red line represents the true trend of diabetes prevalence from 1990 to 2021. Yellow dotted lines and shaded areas represent predicted trends and their 95% CI

The ARIMA model was used to quantitatively describe the trends in the prevalence of diabetes from 2022–2050. After filtering by auto.arima() function, the optimal model for the prevalence of diabetes in China was (2,1,0), with AIC value of 303.75. The optimal model of global prevalence of diabetes was (0,2,0), and AIC value was 259.75. The results show (Fig. 9, Supplementary Table 1) that the ASPR of diabetes in China is expected to increase by 37.19% during 2022–2050, from 6124.19to 8401.66per 100,000(Supplementary Table 1). The global ASPR for diabetes is expected to increase by 69.55%, from 6123.59 to 10382.24 per 100,000 (Supplementary Table 1). In the following three decades, the ASPR of both the world and China showed an upward trend (Fig. 9). The ASPR of different genders showed an upward trend both in China and globally (Supplementary Fig. 5).

## Discussion

This study, based on the GBD 2021 data, systematically analyzed the temporal and spatial evolution characteristics of diabetes burden in China and globally from 1990 to 2021. It was found that the average annual growth rates of the ASIR, ASPR and ASDR of diabetes in China were 1.30, 1.77, and 0.76% respectively. Although they were lower than the global growth rates of 1.74, 2.10, and 1.06% respectively, due to the large population base, the number of patients with diabetes reached 117 million in 2021, accounting for 22% of the global diabetes population [1, 2]. This result is highly consistent with the conclusion in the 2024 GBD report of The Lancet that China is the country with the heaviest diabetes burden in the world, and further reveals that in the period from 1996 to 1999, the ASPR growth rate reached 4.69%, which was significantly related to the annual increase of 1.3% in urbanization rate [1].

The continuous growth of diabetes burden in China can be attributed to the synergistic effect of Westernization of lifestyle and population structure transformation. A study in Diabetes Care in 2023 pointed out that from 1990 to 2021, the annual increase of high-calorie diet consumption among Chinese residents was 4.30%, and the rate of insufficient physical activity reached 31.70%, resulting in an increase in obesity rate from 3.10 to 16.40%, and the risk of diabetes in obese individuals was 3.20 times that of normal-weight individuals [2, 4]. It is worth noting that China's ASMR decreased by 0.24% during the same period, which was related to the popularization of new hypoglycemic drugs and the improvement of grassroots medical capabilities. Globally, an increase of 0.26% in ASMR highlighted the shortcomings in prevention and control in low- and middle-income countries, such as the insufficient availability of insulin in sub-Saharan Africa, resulting in a mortality rate of diabetic ketoacidosis 4.7 times higher than that in high-income regions [1, 9]. The regional differentiation of “lifestyle type”and “aging type”burden in China provided a scientific basis for graded prevention and control.

The prevalence of diabetes in China is expected to reach 84.01 million (ASPR growth of 37.19%) by 2050, posing multiple challenges to public health strategies. From the perspective of prevention strategies, the 2020 Edition of the Chinese Guidelines for the Prevention and Treatment of Type 2 Diabetes pointed out that since diabetes is closely related to lifestyle, vigorous health education should be carried out among the general population, advocating balanced diet and increased physical activity, especially for the middle-aged and elderly population (aged 65–94), measures for community screening and early intervention need to be strengthened [23]. In terms of health resource planning, based on the growth trend of diabetes patients, the number of endocrinology specialist medical resources should be expanded proportionally, such as referring to relevant research recommendations to increase the number of diabetes specialist beds, equip advanced continuous glucose monitoring equipment, and strengthen the professional training of grassroots medical staff to improve diabetes management capabilities [24]. When formulating individualized intervention measures, gender and age differences should be considered. Studies have shown that for men, given that their risk of developing diabetes significantly increases due to unhealthy habits such as smoking and excessive drinking, smokers have a 30–40% higher risk of developing the disease compared to non-smokers. Nicotine reduces muscle glucose uptake and induces insulin resistance [25]. Therefore, a phased smoking cessation plan should be formulated, limiting weekly alcohol intake to ≤ 140 g. At the same time, resistance training three times a week for 30 min each time should be adopted to control waist circumference < 90 cm to manage central obesity, and annual screening for metabolic syndrome should be intensified, paying attention to fasting blood sugar, blood lipids, and blood pressure indicators [26]. For women, considering that the decline in estrogen levels after menopause can lead to reduced insulin sensitivity, the risk of diabetes for postmenopausal women is 1.42 times higher than that of men. It is necessary to assess estrogen levels and, if necessary, adopt local estrogen replacement therapy [27]. Cognitive behavioral therapy should be used to optimize sleep, and a low-GI diet combined with aerobic dancing should be adopted to adjust body fat distribution. In addition, both men and women should follow the Mediterranean diet pattern, ensuring 150–180 min of moderate-intensity exercise and resistance training per week, and manage psychological stress through mindfulness meditation and other methods to reduce the risk of developing diabetes [26]. Moreover, as the impact of aging on the burden of diabetes becomes increasingly prominent, a diabetes prevention and control system covering the entire life cycle should be constructed, integrating resources for geriatric disease prevention and diabetes management [28].

However, this study has some limitations. Firstly, our research is a secondary data analysis of the GBD study. This study inevitably has some shortcomings, such as issues related to data quality assurance; Secondly, the factors influencing the burden of diabetes are complex and diverse. Some potential factors, such as exposure to environmental pollutants and social psychological factors, have not been deeply explored; Thirdly, the predictive model roughly describes future trends, but it cannot provide accurate predictions. In addition, we need to convert the discussion part of this study into practical actions, such as formulating public policies and providing information for subsequent research.

## Conclusions

This study conducted a systematic analysis of the diabetes burden in China and globally from 1990 to 2021 and found that during this period, the incidence, prevalence, and DALYs of diabetes increased in both China and the world. The mortality rate decreased in China while it increased globally. There are age and gender differences in the diabetes burden. The burden is higher in middle—aged and elderly people. Overall, men are more affected, but some indicators are higher in women at advanced ages. Affected by factors such as lifestyle and population aging, it is expected that the prevalence of diabetes in China and the world will continue to rise by 2050. In view of this, China faces severe challenges and needs to strengthen health education, improve the screening and diagnosis mechanism, and increase research investment to effectively prevent and control diabetes and reduce the disease burden.

## Supplementary Information

Supplementary Material 1.

Supplementary Material 2.

## Data Availability

No datasets were generated or analysed during the current study.

## Abbreviations

GBD: Global burden of diseases
DALYs: Disability-adjusted life years
ASIR: Age Standardized incidence rate
ASPR: Age Standardized prevalence rate
ASDR: Age Standardized disability—adjusted life year rate
ASMR: Age Standardized mortality rate
AAPC: Average annual percentage change
APC: Age-period-cohort
ARIMA: Autoregressive integrated moving average
