# Supplementary material for: Trends and comparisons of diabetes burden in China and the world from 1990 to 2021,with forecasts to 2050:a systematic analysis of the global burden of disease study 2021
Source: Diabetol Metab Syndr. 2025 Aug 2;17:309. doi: 10.1186/s13098-025-01885-4 (PMC12317625; doi:10.1186/s13098-025-01885-4)
Supplement: Supplementary file 1 — Supplementary Material 1. [file 13098_2025_1885_MOESM1_ESM.docx]

| 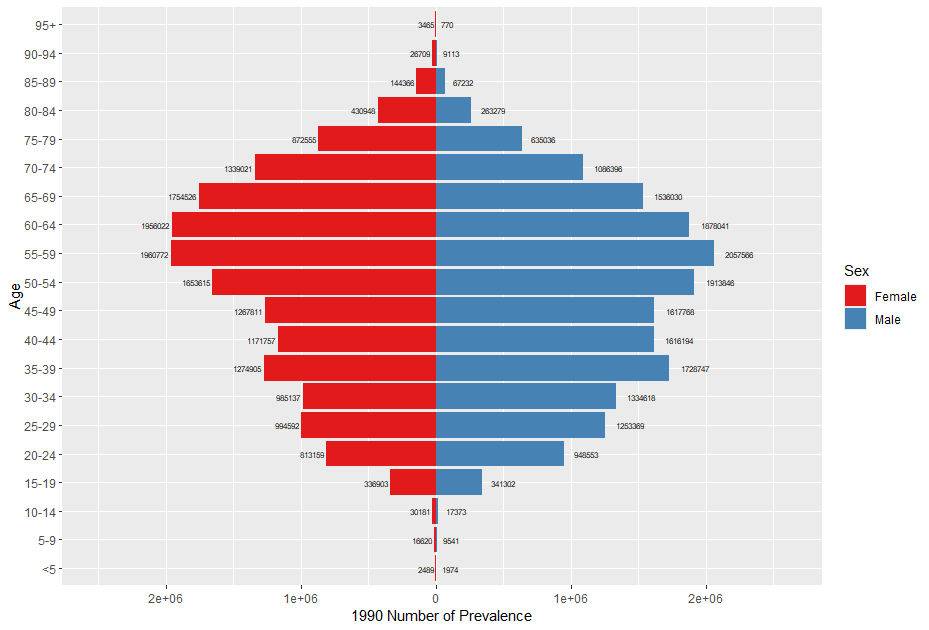 | 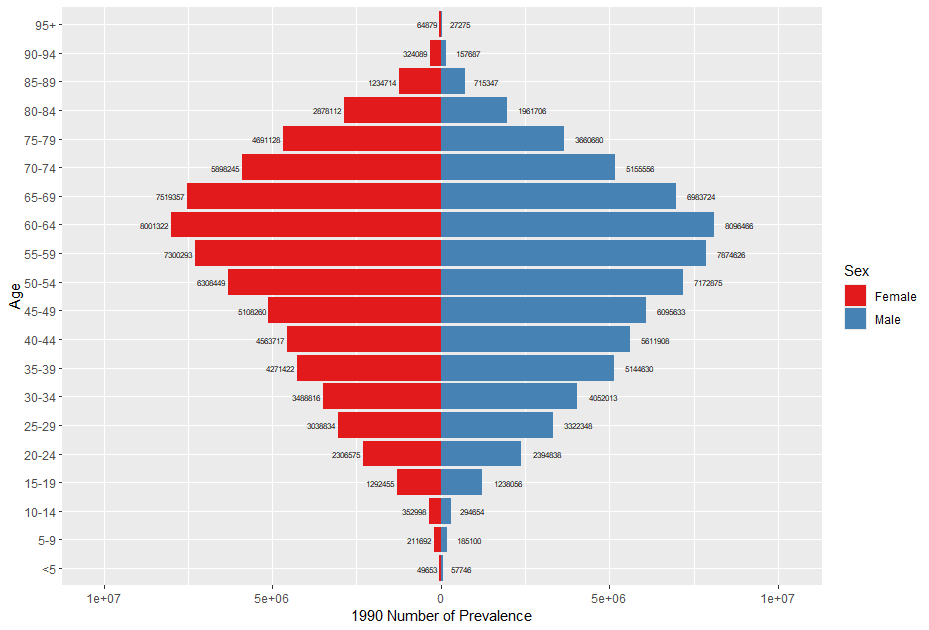 |
| --- | --- |
| (a) | （b) |
| 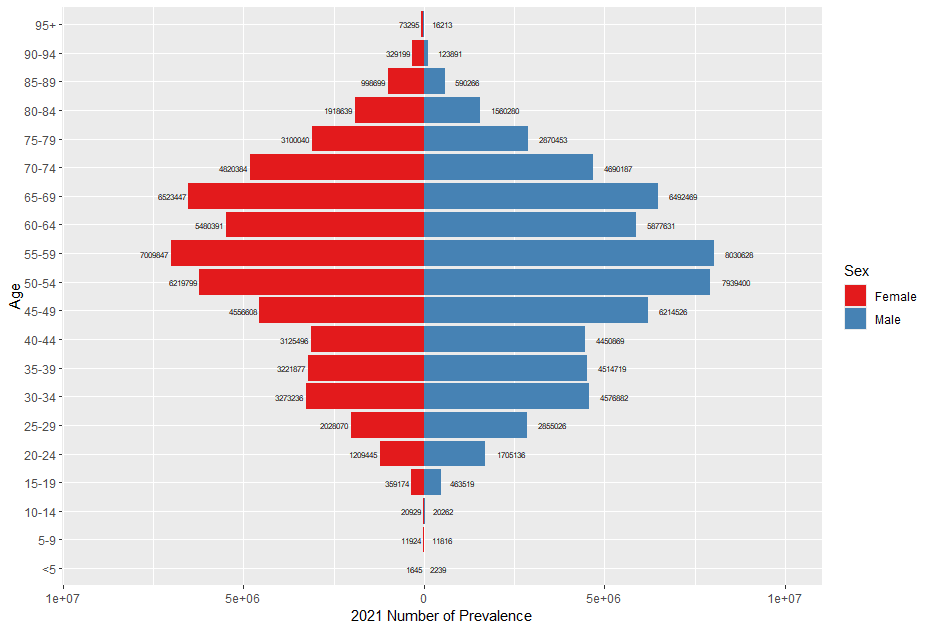 | 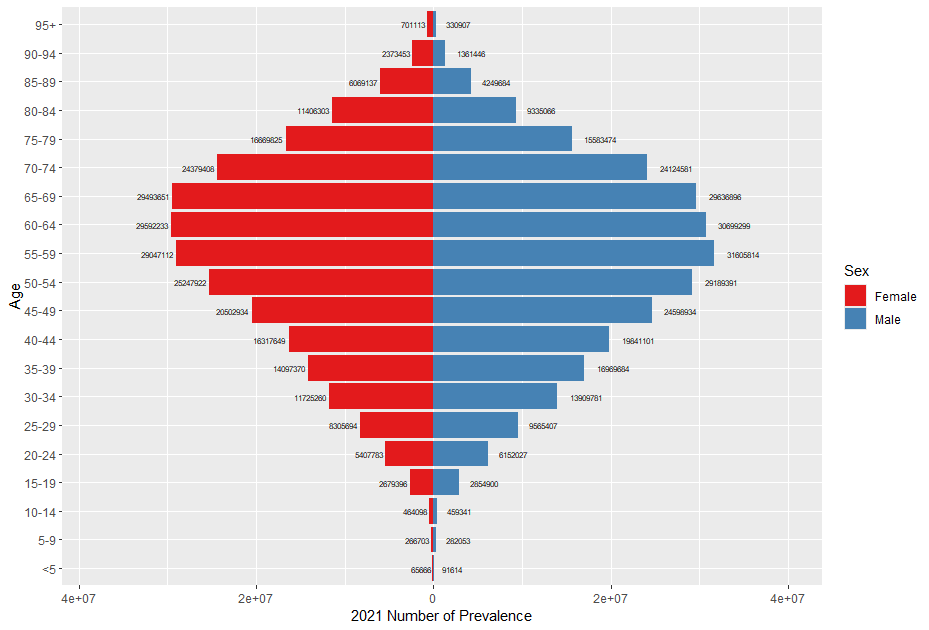 |
| (c) | (d) |
| **Supplementary Fig.1** Prevalence of diabetes in different age groups of men and women in China and the world in 1990 and 2021 (a) prevalence in China in 1990; (b) Global prevalence in 1990; (c) prevalence in China in 2021; (d) Global prevalence in 2021 | |

| 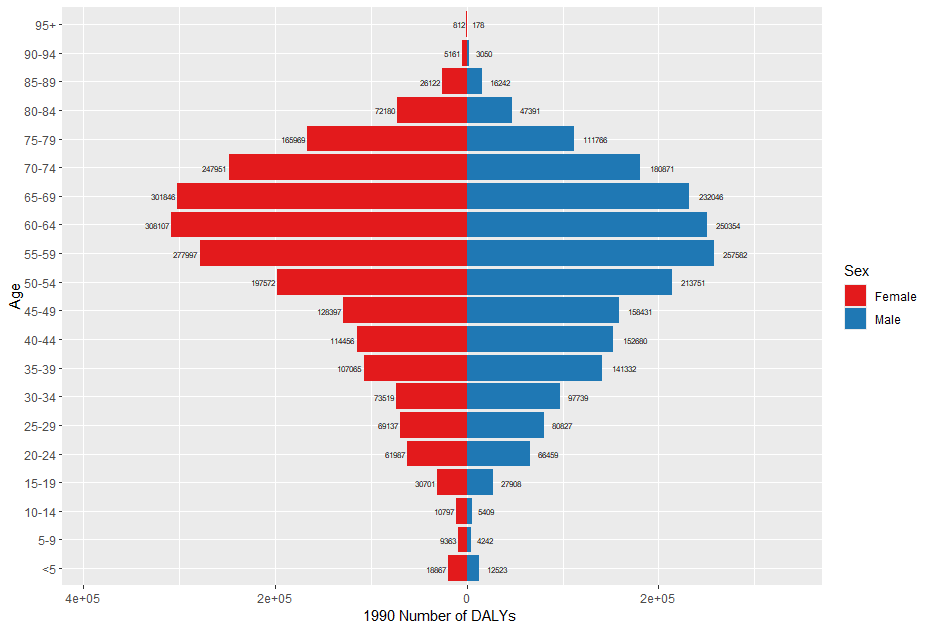 | 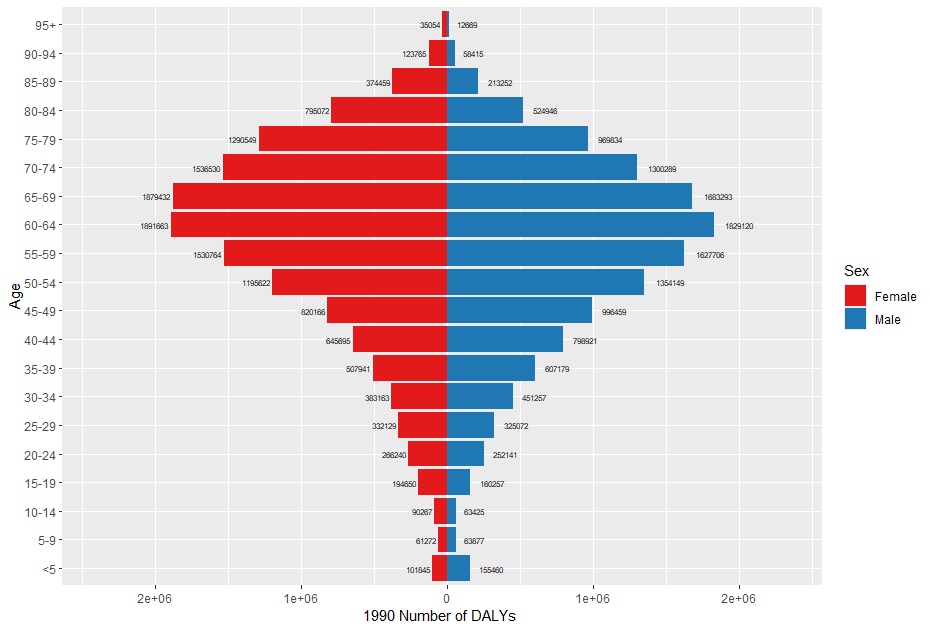 | |
| --- | --- | --- |
| (a) | (b) | |
| 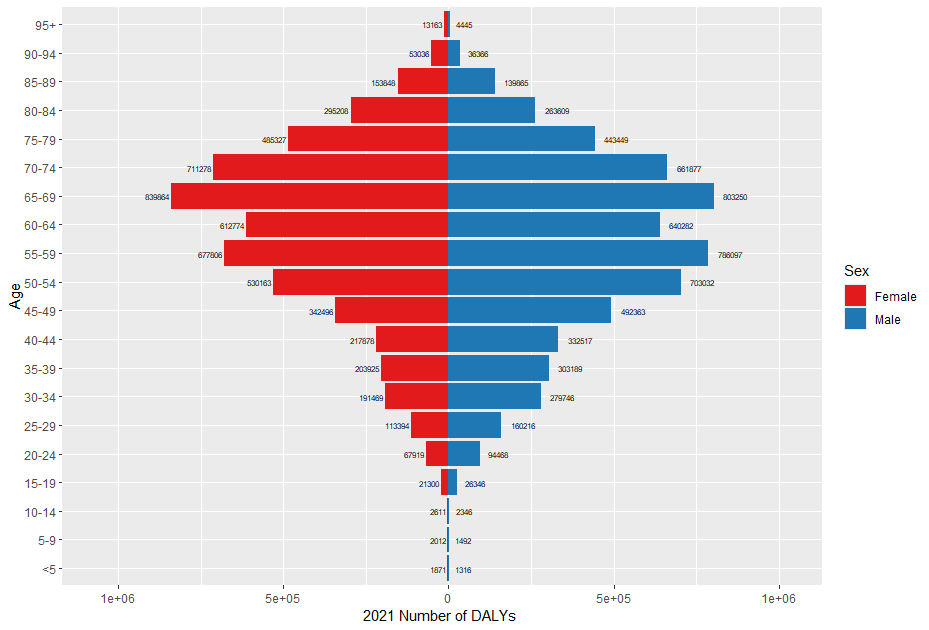 | 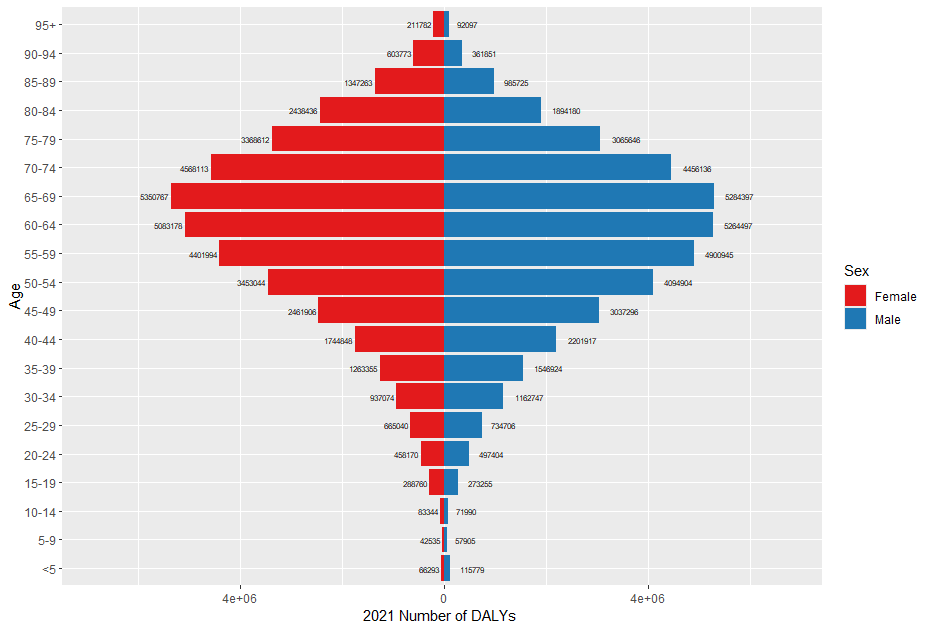 | |
| (c) | (d) | |
| **Supplementary Fig.2**  DALYs of diabetes in different age groups of men and women in China and the world in 1990 and 2021 (a) DALYs in China in 1990; (b) Global DALYs in 1990; (c) DALYs in China in 2021; (d) Global DALYs in 2021 | | |
| 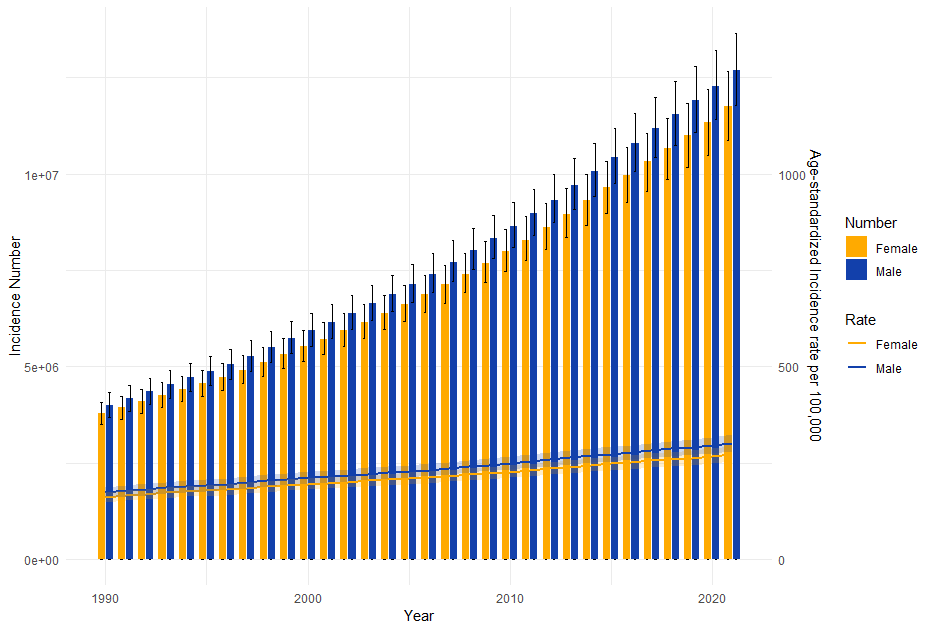 | | 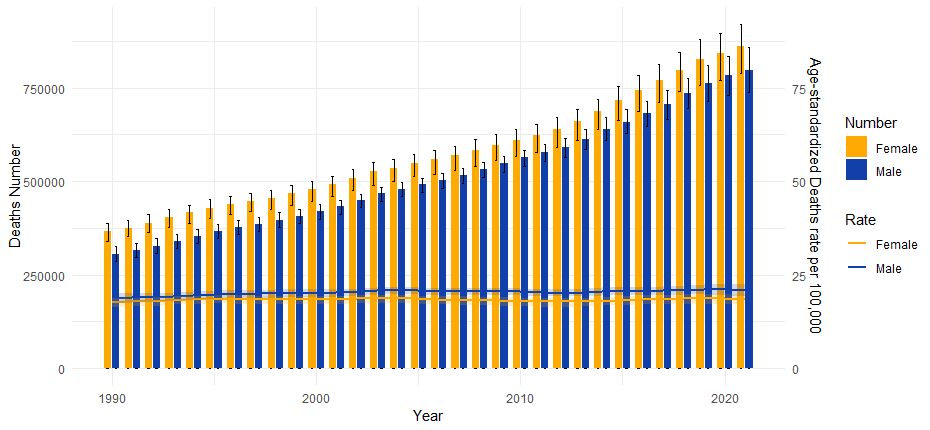 |
| (a) | | (b) |
| 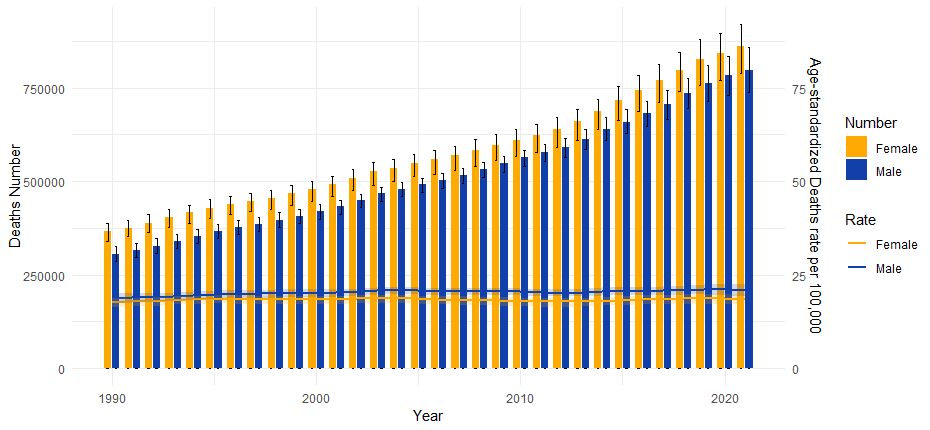 | | 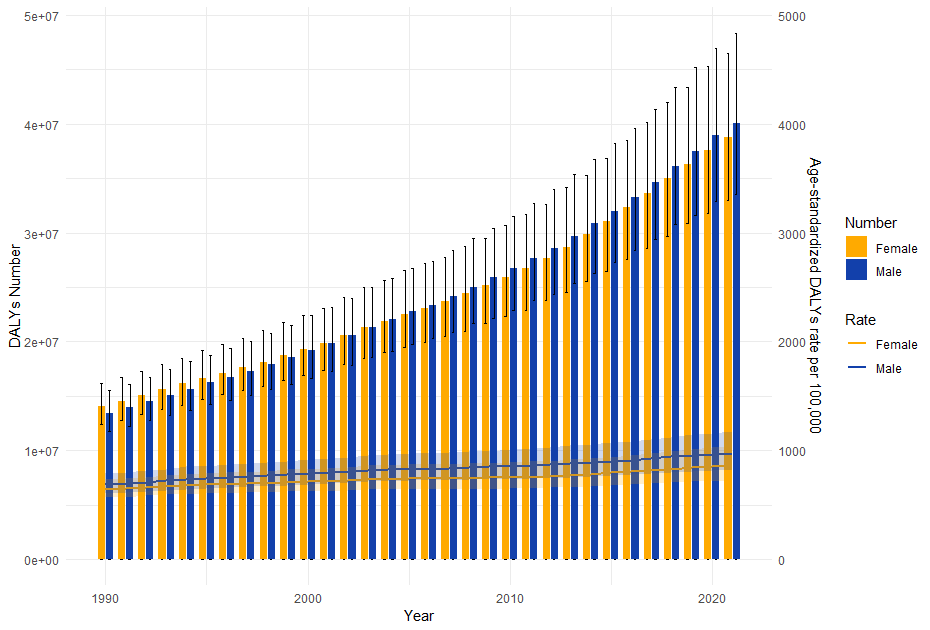 |
| (c) | | (d) |
| **Supplementary Fig.3** Comparison of full-age cases and age-standardized rates of incidence, prevalence, mortality and DALYs among men and women in China from 1990 to 2021. (**a**) Incident cases and ASIR; (**b**) Prevalent cases and ASPR; (**c**) Death cases and ASMR; (**d**) DALYs counts and ASDR. Bar charts represent counts; lines represent age-standardized rates   \| 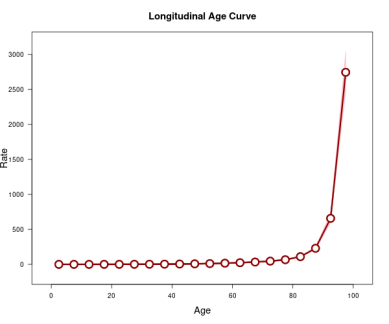 \| 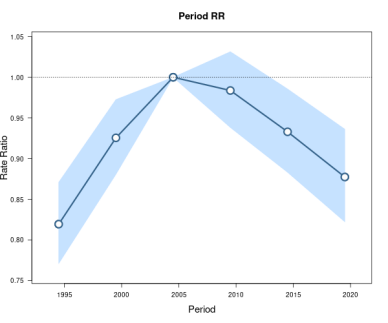 \| 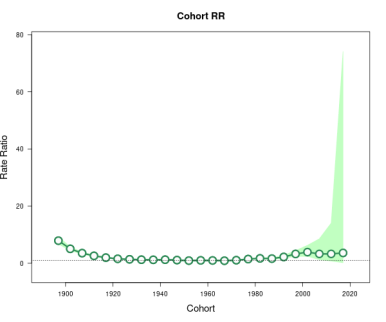 \| \| --- \| --- \| --- \| \| (a) \| (b) \| (c) \| \| 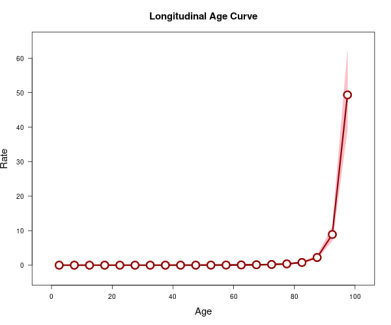 \| 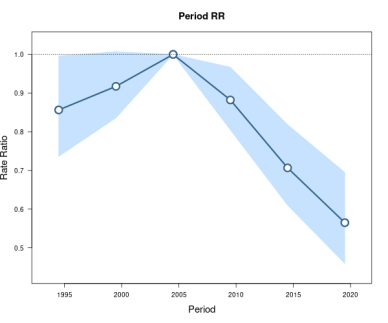 \| 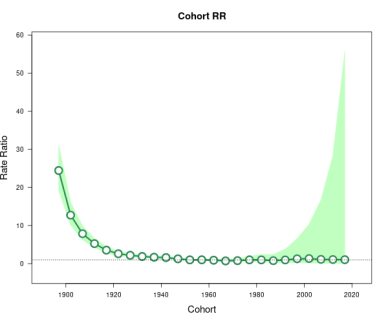 \| \| (d) \| (e) \| (f) \| \| **Supplementary Fig.4** Trends in the prevalence and mortality of diabetes in Global from 1990 to 2021. (a) age effect of prevalence (b) period effect of prevalence (c) cohort effect of prevalence (d) age effect of mortality (e) period effect of mortality (f) cohort effect of mortality \| \| \|  \| 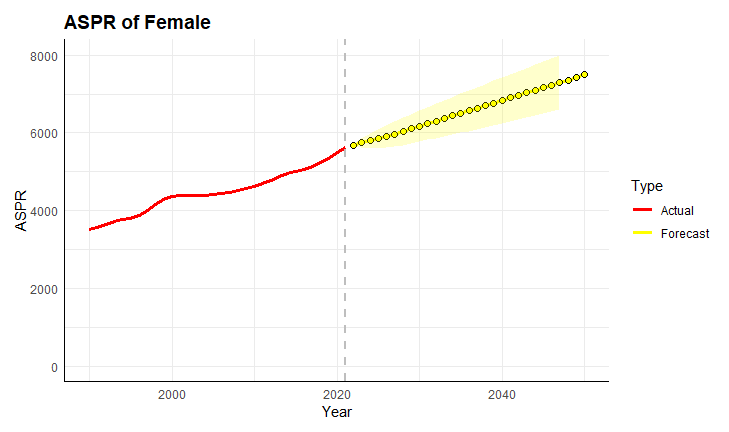 \| 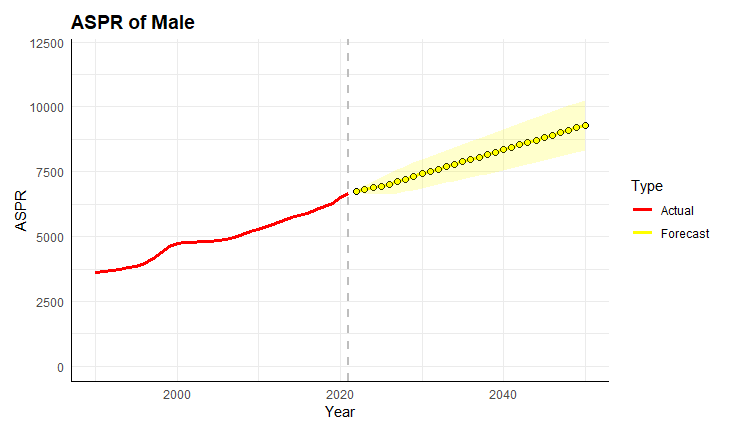 \| \| --- \| --- \| \| (a) \| (b) \| \| 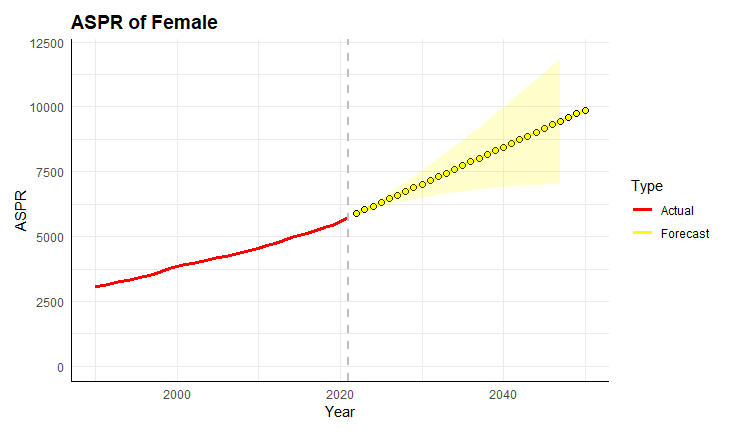 \| 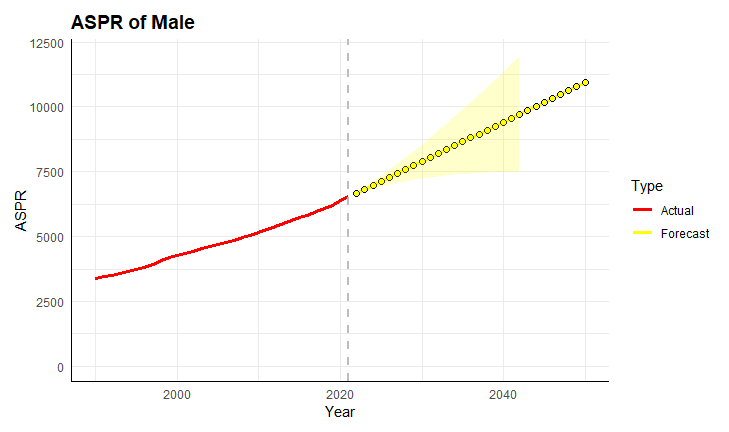 \| \| (c) \| (d) \| \| **Supplementary Fig.5** Prediction of diabetes ASPR by gender in China and the world 2022-2025 (a) ASPR of Chinese female (b) ASPR of Chinese male (c) ASPR of global female (d) ASPR of global male \| \| | | |
|  | | |
|  | | |
